# Indirect Targeting of Subthalamic Deep Brain Stimulation Guided by Stereotactic Computed Tomography and Microelectrode Recordings in Patients With Parkinson’s Disease

**DOI:** 10.3389/fnhum.2018.00470

**Published:** 2018-12-04

**Authors:** Po-Hsun Tu, Zhuo-Hao Liu, Chiung Chu Chen, Wey Yil Lin, Amy L. Bowes, Chin Song Lu, Shih-Tseng Lee

**Affiliations:** ^1^Department of Neurosurgery, Chang Gung Memorial Hospital at Linkou, Chang Gung Medical College and University, Linkou, Taiwan; ^2^Department of Neurology, Chang Gung Memorial Hospital at Linkou, Chang Gung Medical College and University, Linkou, Taiwan; ^3^Neuroscience Research Center, Chang Gung Memorial Hospital, Taipei, Taiwan; ^4^Department of Neurology, Landseed Hospital, Taoyuan, Taiwan; ^5^Royal Free London NHS Foundation Trust, Royal Free Hospital, London, United Kingdom

**Keywords:** subthalamic nucleus, deep brain stimulation, Parkinson’s disease, head frame fixation, effect factor, outcome

## Abstract

**Objective:** Magnetic resonance imaging fusion techniques guided by frame-based stereotactic computed tomography and microelectrode recordings are widely used to target the subthalamic nucleus. However, MRI is not always available. The aim of this study was to determine whether the indirect targeting of the subthalamic nucleus for deep brain stimulation using frame-based stereotactic computed tomography and microelectrode recording guidance in patients with advanced idiopathic Parkinson’s disease was an effective and safe treatment and to determine the factors that contributed to outcome.

**Methods:** Thirty-four consecutive patients with Parkinson’s disease who were treated from 2010 to 2012 were enrolled in this retrospective cohort study. The patients were assessed with the Unified Parkinson’s Disease Rating Scale-part III (UPDRS-III) and other clinical profiles peri- and post-operatively. The horizontal and vertical distances between the midpoint of the head frame and the brain midline at the septum pellucidum level and the upper edge of the bilateral lens, respectively, on a thin-section brain computed tomography scan were defined as the horizontal and vertical deviations, respectively.

**Results:** After the deep brain stimulation surgery, the patients’ UPDRS-III scores improved 48 ± 2.8% (range, 20–81%) compared to the patients’ baseline off-levodopa scores. No surgery-associated complications were found. The mean recorded length difference of the subthalamic nucleus between the initial and final single microelectrode recording trajectories was 5.37 ± 0.16 mm (range, 3.99–7.50). Multiple linear regression analyses revealed that the increased lengths of the vertical (regression coefficient [B]: -0.0626; 95% confidence interval [CI]: -0.113 to -0.013) and horizontal deviations (B: -0.0497; 95% CI: -0.083 to -0.017) were associated with less improvement in the patients’ UPDRS scores.

**Conclusion:** These results showed that the indirect targeting of the subthalamic nucleus for deep brain stimulation using frame-based stereotactic computed tomography and microelectrode recording guidance in patients with advanced idiopathic Parkinson’s disease was effective and safe. Greater symmetry of the head frame fixation resulted in better outcomes of the deep brain stimulation of the subthalamic nucleus in patients with Parkinson’s disease, especially when the horizontal deviation was 2 mm or less and the vertical deviation was 1 mm or less.

## Introduction

The benefits of deep brain stimulation (DBS) of the subthalamic nucleus (STN) in patients with Parkinson’s disease (PD) have been well-defined (Bejjani et al., 2000; Kleiner-Fisman et al., 2003; Krack et al., 2003; Rodriguez-Oroz et al., 2005; Deuschl et al., 2006; Erola et al., 2006; Benabid et al., 2009; Gervais-Bernard et al., 2009; Weaver et al., 2009). Under strict selection criteria for candidates, the clinical efficacy of STN-DBS relies on correct targeting of the STN through different surgical strategies (Cuny et al., 2002; Hickey and Stacy, 2016). Several targeting methods are used in DBS, and the appropriate method is selected depending on the familiarity of the neurosurgeon with the procedure, the condition of the patient, and the facilities of the institution. Many methods, such as magnetic resonance imaging (MRI) direct targeting, image fusion techniques involving co-registered computed tomography (CT) and MRI, and indirect targeting using intraoperative ventriculography have been used to accurately locate DBS targets.

Although intraoperative ventriculography provides reliable identification of the anterior commissure (AC) and posterior commissure (PC), which are landmarks for indirect targeting methods, it is currently used less in most practicing centers. In contrast to ventriculography, CT and MRI are less invasive and more easily performed. Because of the distortional visualization of the STN with MRI and the poor visualization of the AC-PC and STN with CT, the fusion of stereotactic CT and MRI methods are thought to combine the advantages of both methods, and increase spatial validity and localization accuracy (Chen et al., 2011). In addition to image targeting, microelectrode recordings (MERs) are used with DBS to ensure the optimal placement of DBS electrodes along the subthalamic homunculus (Romanelli et al., 2004).

However, performing MRI on patients with advanced PD may be difficult and even contraindicated due to their symptoms, including severe dystonia, obvious tremor, severe stoop posture, and claustrophobia. Therefore, instead, we have used frame-based stereotactic CT and MERs to target the STN at our hospital. Indirect targeting with CT is used to locate the STN, while MERs are used to determine the optimal trajectory for accurate placement of the DBS electrodes along the subthalamic homunculus. This study was performed to investigate whether the use of frame-based stereotactic CT and MERs was accurate for guiding the targeting of the STN in DBS and the factors that contributed to outcome. Due to the indirect targeting, we especially focused on whether the symmetry of the head frame fixation directly affected the patients’ motor improvements.

## Materials and Methods

### Patient Selection

Approval for this retrospective cohort study was granted by the Institutional Review Board of Chang Gung Memorial Hospital in Taiwan (IRB.105-039C). Thirty-four patients with idiopathic and advanced PD who underwent DBS of the bilateral STN in our hospital were recruited for the study from June 2010 to December 2012. Each patient participated in a minimum follow-up period of 5 years (range, 60–83 months) in our outpatient clinic.

The diagnosis of idiopathic PD was made according to the United Kingdom Brain Bank criteria for symptoms that were present for at least 5 years (Hughes et al., 1992). Patients were diagnosed with advanced PD if they presented at least one of the following: (1) on-off phenomenon, (2) increased off period, (3) severe off-period dystonia, (4) intractable tremor motor fluctuations, or (5) drug-induced dyskinesia (Okun, 2012).

Preoperatively, the patients were assessed with the Unified Parkinson’s Disease Rating Scale-part III (UPDRS-III), Modified Hoehn and Yahr Scale, Schwab and England Activity of Daily Living (ADL) scale, Beck Depression Inventory, Mini-Mental State Examination, Clinical Dementia Rating, and basic neuropsychological tests to exclude moderate to severe dementia, anxiety, and depression, and positron emission tomography was used to differentiate PD from other disorders. We performed the surgery if the patient showed a greater than 30% improvement on the UPDRS-III after the levodopa challenge test, and we excluded patients with Parkinsonism and any cognitive and psychiatric problems, which were determined by a neurologist and psychologist.

### Operation

**FIGURE 1:**
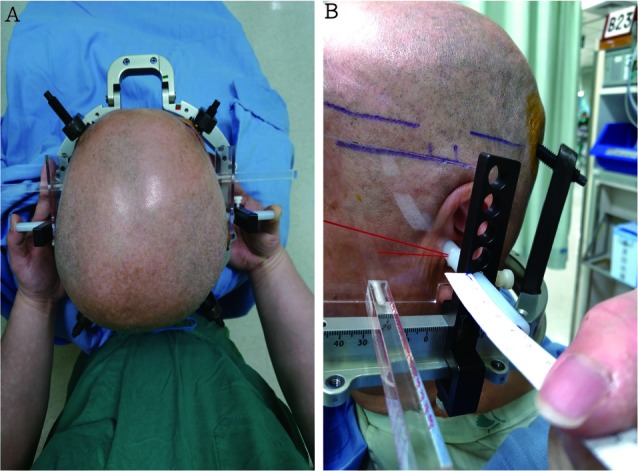
**(A)** An ear bar, which was used to balance the ideal position of the frame while applying the pins, was utilized to assist head frame fixation and decrease the vertical and horizontal deviations between both sides. **(B)** The head frame was placed so that it was centered on the midline (correct by ruler), thus allowing for maximal symmetry between the right and left sides and so that it was as parallel as possible to the anterior commissure and posterior commissure – right to left symmetry plane (The picture is permitted for use by the patient).

The patients underwent STN DBS following levodopa withdrawal the night before the surgery. In addition, dopamine agonists were withheld for 48 hours. Before fixation with the Cosman-Roberts-Wells frame (Integra Radionics, Burlington, MA, United States) fixation, we marked the insert points of the fixed-frame screws on the patient’s head. An ear bar was used to assist fixation of the head frame by balancing the frame in the ideal position while applying the pins and decrease the vertical and horizontal deviations between both sides. Under local anesthesia, the head frame was positioned so that it was centered on the midline to allow for the maximum symmetry between the right and left sides so that it was as parallel as possible to the AC to PC and right to left symmetry planes (Figure 1). A stereotactic 1-mm-thick, section-angle parallel to frame, non-enhanced CT image was obtained using a CT scanner. Subsequently, these Digital Imaging and Communication in Medicine images were transferred to a workstation (Brainlab AG, Munich, Germany) for target planning.

A preliminary indirect target was selected for stereotactic CT based on typical brain atlas coordinates of the target structure (Schaltenbrand and Wahren, 1977; Kleiner-Fisman et al., 2003). The intended coordinates for the target point were 12 mm lateral from the midline, 3 mm behind the midcommissural point, and 4–5 mm below the AC-PC line. The safe transfrontal lobe trajectory to the target was set approximately 15 degrees from the sagittal plane and 60–70 degrees in the anterior-posterior direction while avoiding cortical and periventricular veins, sulci, and ventricles (Machado et al., 2006). Physiological confirmation and fine-tuning of the target region was confirmed using MERs (LeadPoint work station; Medtronic, Minneapolis, MN, United States). Approximately reaching the STN, the MER activity showed increased amplitude, which corresponded to the STN. When the electrode entered the STN, background activity increased sharply, and high-amplitude irregular spikes with firing rates between 30 and 70 Hz appeared. The exit of the tip of the electrode out of the STN was indicated by a decrease in background noise and the incidence of spontaneous neuronal activity compared with when the electrode was in the STN. The MERs were terminated when neuronal activity was encountered that was typical of the substantia nigra, which was characterized by regular discharges at 70–110 Hz. The lower border of contact 0 was implanted in the substantia nigra reticulata such that contact 2 or 3 was likely located in the dorsal sensorimotor area of the STN. Entry into and exit out of the STN was determined by two authors (CC and WL). The unilateral length of the STN was the distance between the MER entry into and exit out of the STN (Benazzouz et al., 2002). We stopped the procedure if the unilateral length of the STN determined by the MERs was less than 4 mm after two trajectories on each side. However, the procedure was successful in all 34 patients. Intraoperative bipolar test stimulations were performed using an external stimulator (Medtronic dual screen 3625) to test for clinical or side effects upon insertion of the DBS electrode. The stimulation was performed using each of the three contact pairs (01, 12, and 23). The stimulation voltage was gradually increased to 3–3.5 V. The macroelectrode was the 3389 model (Medtronic), which has four platinum-iridium cylindrical surfaces (1.27 mm in diameter and 1.5 mm in length). Immediately after the surgery, all patients underwent T2-weighted MRI to confirm that at least one DBS contact was in the STN region. The electrodes were connected to a subcutaneous programmable pulse generator (Kinetra 7428; Medtronic) 5 days after the initial implantation. All patients were administered monopolar stimulation and pulses with individually set parameters with ranges of 2.4–4.3 V, 60 μs, and 80–180 Hz.

### Patient Evaluations

All patients were evaluated by two authors (CC and WL). The preoperative clinical details of the patients are summarized in Table 1.

**Table 1:** Patient demographics and PD characteristics.

Variable	Mean SEM or frequency (percentage)
Patient number	34
Age (years)	58.9 ± 1.6
Male sex, n (%)	23 (68)
Disease duration (years)	14.6 ± 0.9
Preoperative, UPDRS- I, medication off	2.94 ± 0.8
Preoperative, UPDRS-II, medication off	20.28 ± 0.38
Preoperative, UPDRS-III, medication off	48.9 ± 1.7
Preoperative MMSE	26.48 ± 0.47
Preoperative CDR	0.29 ± 0.05
Preoperative depression rating scale	2.06 ± 0.56
Preoperative levodopa response (%)	46 ± 3
Preoperative Hoehn and Yahr stage, medication off	3.36 ± 0.12
Preoperative ADL, medication off	50.76 ± 4.41
Improvement rate (%)	48 ± 2.8

*ADL, activities of daily living; PD, Parkinson’s disease; UPDRS, unified Parkinson’s disease rating scale; MMSE, mini-mental state examination; CDR, clinical dementia rating. The data are presented as mean ± standard error of the mean (SEM) for continuous variables or frequency (percentage) for categorical variables.*

Thirty-six months after the surgery, the patients were assessed with the UPDRS-III after overnight withdrawal of anti-parkinsonian medications. The difference in the postoperative UPDRS-III scores of the patients between the off and on DBS stimulations during the off medication state was defined as A. The preoperative baseline UPDRS-III score in the off medication state was defined as B. The rate of UPDRS-III score improvement was defined as A/B × 100 (Obeso et al., 2001).

**FIGURE 2:**
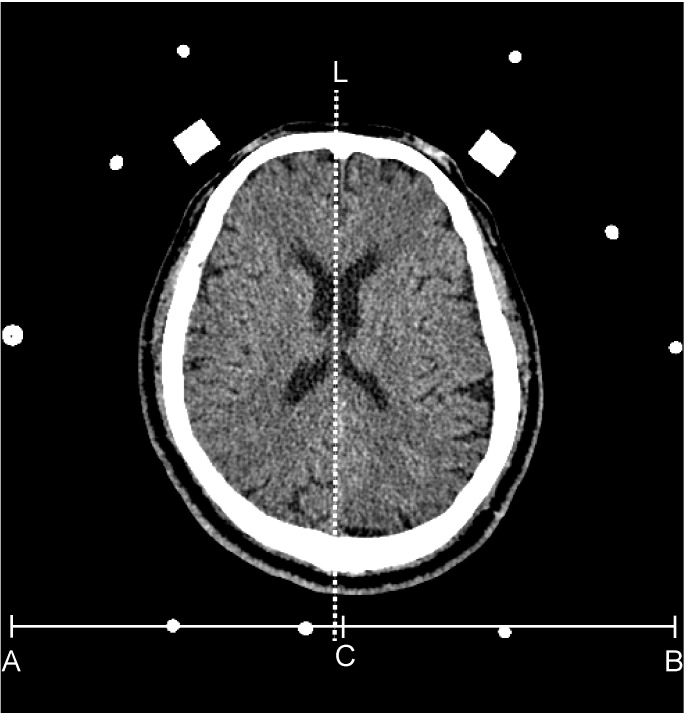
The dash line (L) indicates the position of the septum pellucidum in an axial view of a brain CT. The C point is the mid-point of the x-axis (from A to B) of the head frame. The distance between the dash line and point C is defined as the horizontal deviation.

**FIGURE 3:**
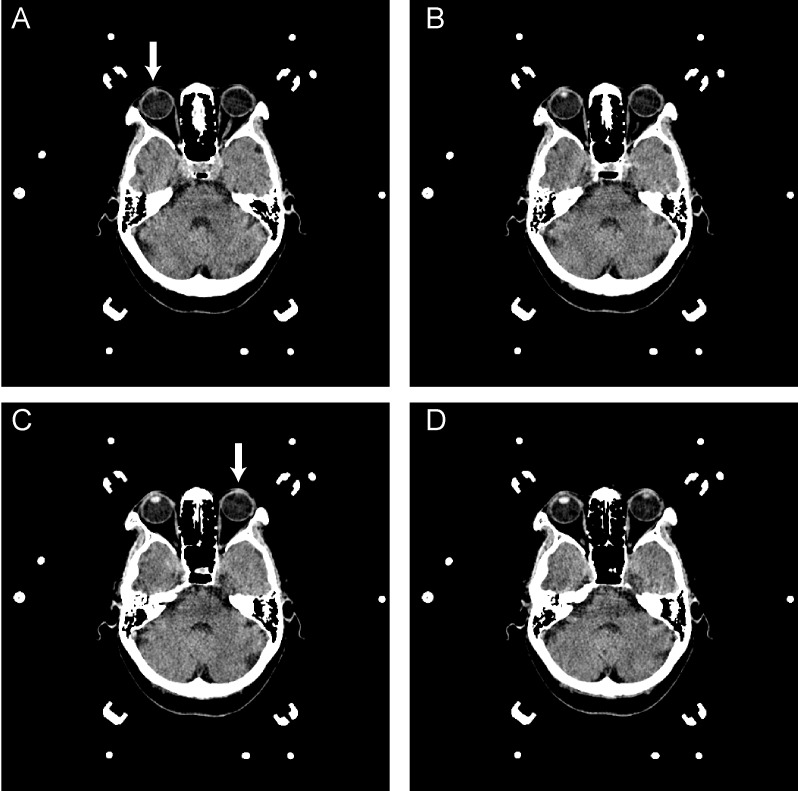
**A–D** are serial axial images of a brain CT for DBS surgery. The interval distance of the serial image is 1 mm. **A** reveals the upper edge of the right lens (arrow); however, the left lens was unavailable in this image. In **C**, the upper edge of the left lens was presented, as indicated by the arrowhead. The vertical deviation was calculated from the serial image as 2 mm.

The CT images of septum pellucidum close to the AC-PC line were chosen for analysis for horizontal deviation. The horizontal distance between the midpoint of the head frame and the midline of the brain on a thin CT scan section (slice thickness: 1 mm) at the septum pellucidum level was evaluated preoperatively and defined as the horizontal deviation (Figure 2). The vertical distance of the upper edge of the bilateral lens on a thin CT scan section (slice thickness: 1 mm) was evaluated preoperatively and defined as the vertical deviation (Figure 3). The Stealth Station S7 Surgical Navigation System (Medtronic) was used to detect pneumocephali on postoperative MRI scan, which were checked to confirm that at least one DBS contact was in the STN region. The length of the STN was defined as the average of the distances between the entry and exit points of the bilateral STN, which was determined using MERs. The size of the STN was reported using the anteroposterior (mean, 5.9 mm), mediolateral (3.7 mm), and dorsoventral (5 mm) dimensions (Richter et al., 2004) The entrance trajectories of the MERs into the STN in the anterolateral direction therefore determined that the appropriate length of the STN was between 4 and 6 mm.

### Statistical Analysis

The statistical and receiver operating characteristic (ROC) curve analyses were conducted using GraphPad Prism 6 (GraphPad Software, La Jolla, CA, United States) and MedCalc 13 (MedCalc Software bvba, Ostend, Belgium). Continuous variables are reported as the mean ± standard error of the mean (SEM), while categorical variables are presented using frequencies and percentages. Group differences (favorable outcomes vs. unfavorable outcomes) were compared using two-sample *t*-tests for continuous variables or chi-square test for categorical variables. To identify factors that were potentially associated with the improvements in the UPDRS scores, a series of univariate linear regression analyses were performed (data not shown). Variables with *p*-values less than 0.2 were then introduced, along with sex and age, into multiple linear regression analyses. (Maldonado and Greenland, 1993; Vittinghoff, 2012) Finally, the ROC curves were analyzed to discriminate the dichotomous outcomes according to the lengths of the vertical and horizontal deviations. A two-tailed α level less than 0.05 was used to determine significance for all statistical tests.

## Results

The general characteristics of the 34 subjects examined in this study are summarized in Table 1. No intracranial hemorrhages were found in the MRI scans performed immediately postoperatively. The mean ± SEM rate of improvement in the UPDRS-III scores was 48 ± 2.8% (range, 20–81). The mean ± SEM length of the STN according to the MERs was 5.37 ± 0.16 mm (range, 3.99–7.50). The mean ± SEM bilateral stimulation parameters of voltage, frequency, and pulse width were 3.30 ± 0.06, 135.48 ± 4.93, and 60 ± 0, respectively. The mean ± SEM volume of the pneumocephali in the postoperative MRIs was 5.29 ± 2.02 c.c. (range, 0–59). The mean ± SEM horizontal deviation was 1.7 ± 1.2 mm (range, 0–5 mm), and the mean ± SEM vertical deviation was 1.2 ± 0.7 mm (range, 0–2 mm).

Because we do have a standard for grading outcome and the mean improvement rates on the UPDRS-III scores in Taiwan are about 40–51.37% (Chen et al., 2011; Jiang et al., 2015), we divided the patients into the following two groups according to the mean rate of improvement on the UPDRS-III in this study: favorable (motor improvement ≥ 48%) or unfavorable (motor improvement < 48%). The mean ± SEM percentages of improvement in the favorable and unfavorable groups were 63.31 ± 0.03 (range, 49–81) and 36.39 ± 0.02 (range, 20–47), respectively. The two groups differed significantly for mean stimulation voltage, improvement rate, vertical deviation, horizontal deviation, and STN length within 4–6 mm (*p* < 0.05). No significant differences were observed in the preoperative motor scores, preoperative Hoehn and Yahr scale scores, preoperative ADL scores, sex, age at the time of the surgery, disease duration, average current, and frequency of the bilateral stimulation, and pneumocephali volume (Table 2).

**Table 2:** Comparison of the favorable and unfavorable groups.

Variable	Favorable outcome	Unfavorable outcome	*p*-value
Patient number	18	16	
Age (years)	60.13 ± 1.85	56.92 ± 2.58	0.602
Male sex, n (%)	13 (72)	10 (63)	0.822
Disease duration (years)	15.07 ± 1.12	13.94 ± 1.48	0.335
Preoperative Hoehn and Yahr stage, medication off	3.41 ± 0.17	3.34 ± 0.20	0.750
Preoperative ADL, medication off	52.35 ± 5.59	48.57 ± 5.93	0.904
Preoperative UPDRS-III, medication off	47.65 ± 2.04	49.57 ± 3.35	0.612
Preoperative levodopa, response (%)	47.65 ± 2.3	45.2 ± 5.4	0.45
Average stimulation parameter: voltage	3.16 ± 0.07	3.50 ± 0.09	< 0.05
Average stimulation parameter: Frequency	135.49 ± 5.28	135.27 ± 8.59	0.116
Average stimulation parameter: pulse width	60 ± 0	60 ± 0	1.00
Improvement rate (%)	63.31 ± 0.03	36.39 ± 0.02	<0.001
Vertical deviation (mm)	0.75 ± 0.13	1.79 ± 0.12	<0.001
Horizontal deviation (mm)	1.24 ± 0.22	2.36 ± 0.38	0.014
STN length within 4–6 mm, n (%)	18 (100)	9 (56)	<0.001
Pneumocephalus volume	5.49 ± 3.64	5.09 ± 1.81	0.720

*ADL, activities of daily living; STN, subthalamic nucleus; UPDRS, unified Parkinson’s disease rating scale. The data are presented as mean SEM for continuous variables or frequency (percentage) for categorical variables.*

The univariate linear regression analyses showed that preoperative UPDRS score, disease duration, levodopa response, STN length within 4–6 mm, vertical deviation, horizontal deviation, and pneumocephali volume were potential explanatory variables (*p* < 0.20) of the improvements in the UPDRS scores (data now shown). The multiple linear regression analyses revealed that the increased lengths of the vertical deviation (Regression coefficient [*B*]: -0.0626; 95% confidence interval [CI]: -0.113 to -0.013) and horizontal deviation (*B*: -0.0497; 95% CI: -0.083 to -0.017) were associated with less improvement on the UPDRS scores (Table 3).

**Table 3:** Multiple linear regression with improvement rate in UPDRS score as the dependent variable in 34 patients.

Explanatory variable	Regression coefficient (*B*)	95% CI of *B*	*p*-value
Age (years)	-0.0013	-0.006, 0.003	0.540
Male sex	-0.0487	-0.118, 0.020	0.156
Preoperative UPDRS-III	0.0009	-0.002, 0.004	0.568
Disease duration (years)	-0.0038	-0.011, 0.003	0.261
Preoperative levodopa response (%)	0.0762	-0.110, 0.263	0.405
STN length within 4–6 mm	-0.0987	-0.210, 0.013	0.079
Vertical deviation (mm)	-0.0626	-0.113, -0.013	0.017
Horizontal deviation (mm)	-0.0497	-0.083, -0.017	0.003
Pneumocephalus volume (c.c)	0.0017	-0.001, 0.005	0.267

*CI, confidence interval; STN, subthalamic nucleus; UPDRS, unified Parkinson’s disease rating scale.*

**FIGURE 4:**
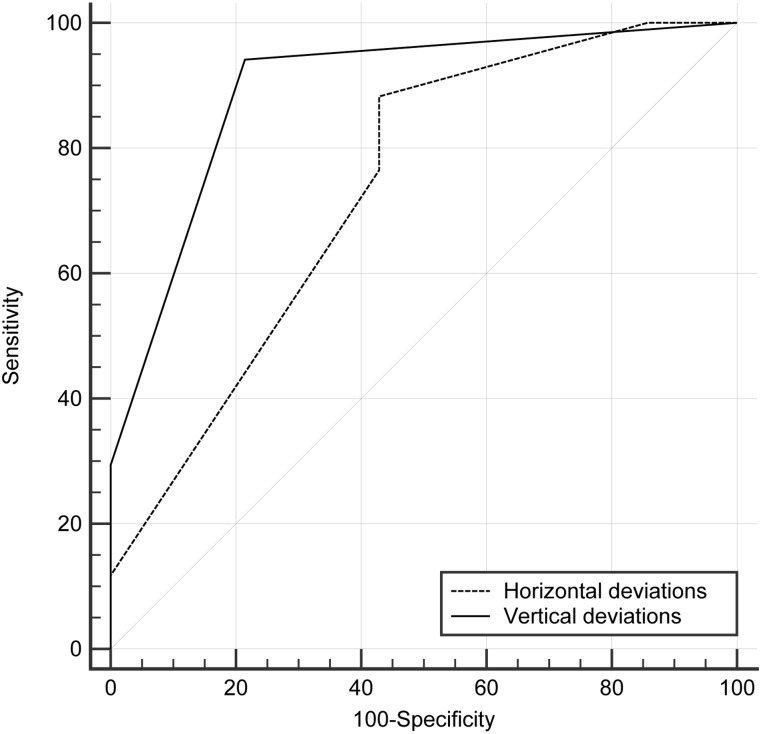
Receiver operating characteristic curve (ROC) analysis of horizontal and vertical deviations in discriminating dichotomous outcome. The area under the curve was 0.74 (95% confidence interval [CI], 0.55–0.88) for horizontal deviations and 0.90 (95% CI, 0.73–0.98) for vertical deviations. The optimal cut-off points for horizontal deviations was ≤ 2 mm with a sensitivity of 88.2 (95% CI, 63.6–98.5) and a specificity of 57.1 (95% CI, 28.9–82.3); and that for vertical deviations was ≤ 1 mm with a sensitivity of 94.1 (95% CI, 71.3–99.9) and a specificity of 78.6 (95% CI, 49.2–95.3).

The ROC curve analyses were performed to evaluate the cut-off points for the vertical and horizontal deviations for discriminating the dichotomous outcome. The analysis revealed that the horizontal and vertical deviations corresponded to areas under the curve (AUCs) of 0.74 (95% CI, 0.55 to 0.88) and 0.90 (95% CI, 0.73 to 0.98). The optimal cut-off points according to the Youden index for the horizontal and vertical deviations were ≤ 2 mm with a sensitivity of 88.2 (95% CI, 63.6 to 98.5) and specificity of 57.1 (95% CI, 28.9 to 82.3) and ≤1 mm with a sensitivity of 94.1 (95% CI, 71.3 to 99.9) and specificity of 78.6 (95% CI, 49.2 to 95.3), respectively (Figure 4).

## Discussion

### Targeting Method and Typical Error During the DBS Surgery

Accurate implantation of depth electrodes into the brain is the greatest importance in DBS, and various stereotactic systems have been developed for electrode implantation (Li et al., 2016). In addition to the widespread use of frame-based stereotactic systems for electrode implantation, frameless, and interventional MRI systems have been used increasingly in recent years (Li et al., 2016). MERs are a neurophysiological method used for target verification before definitive implantation of the DBS lead (Romanelli et al., 2004), and intraoperative recordings of local field potentials have been used to help localize the STN in DBS surgery (Chen et al., 2006; Pearson et al., 2017). Direct image-guided surgeries that do not involve MERs are being increasingly performed, and their targeting accuracies and clinical efficacies are comparable to those involving MERs (Starr et al., 2014; Li et al., 2016). MRI and image fusion techniques involving co-registered CT scanning and intraoperative ventriculography have been used for imaging for DBS surgeries.

Although electrode implantations can be performed with higher accuracy over time (Li et al., 2016), electrode implantation errors can occur in all of the procedures, including fiducial/frame application, imaging and planning, burr hole opening, electrode implantation, and lead fixation (Abosch et al., 2013). Because targeting accuracy is affected by various surgical steps, every step needs to be thoroughly examined to decrease any potential errors.

In this study, we showed that the old technique of the indirect targeting of the STN for DBS that was guided by frame-based stereotactic CT and MERs in patients with advanced idiopathic PD was effective and safe treatment. We also examined whether symmetrical head frame fixation predicted the motor outcomes of patients with PD who underwent frame-based stereotactic CT-guided STN-DBS. We used these measures because they are fast and simply acquired in all patients using frame-based stereotaxic surgery. Fortunately, we found that symmetrical head frame fixation was associated with the amount of improvement in the UPDRS scores in the patients with PD undergoing STN-DBS. The horizontal and vertical deviations of the head frame fixation, as well as the average length of the STN (between 4 mm and 6 mm according to the MERs), were essential for motor improvement after the STN-DBS surgery. However, no predictive values were found for other factors, such as the ADL scale score, Hoehn and Yahr scale score, sex, age, or disease duration.

### Reliable and Time-Saving CT Scans

Previous clinical studies have shown that MRI causes non-linear distortion effects during stereotactic procedures (Burchiel et al., 1996; Chen et al., 2011), while CT is a reliable technique. In frame-based stereotactic STN targeting, an imaging fusion technique involving stereotactic CT and MRI records significantly longer STN lengths through the use of limited MERs compared with MRI alone, but this difference does not result in better clinical outcomes or decreased morbidity (Chen et al., 2011). In our study, the clinical outcomes of the patients that underwent STN-DBS with frame-based stereotactic CT guidance and MER targeting were similar to the patients who underwent STN-DBS with image fusion techniques involving stereotactic CT and MRI (Chen et al., 2011). Performing MRI on patients with advanced PD, especially those in the off-medication state or those who have severe dystonia, obvious tremor, severely stooped posture, or claustrophobia is difficult. The use of long-term sedatives during the MRIs is risky for these patients. However, performing CT scans is much more time efficient, especially when the equipment is a high-speed multi-slice spiral CT scanner. In this study, the stereotactic scan acquisition time was less than 3 min, which was far less than the average MRI acquisition time of 20 min.

### Symmetrical Head Frame Fixation Avoids Coordinate Distortion

The frame is the foundation of frame-based stereotaxy. Perioperative target planning is based on the calculations of the coordinates from the AC and PC, whereas the trajectories are evaluated according to the relationships among the target, insert point, right to left AC-PC symmetry plane, and plane vertical to the right to left AC-PC symmetry plane. When the head-frame was placed symmetrically between the right and left sides as well as centered at the midline, the transformation of coordinates (x, y, z) at the bilateral insert points and targets varied less. In other words, the midline and cross-section of the frame system were parallel to the left to right AC-PC symmetry plane while taking into account the anatomical variations among patients. The rounding of several measures in the three directions to the nearest millimeter when the insert points and targets were calculated might have increased distortion.

### Cut-Off Values of the Horizontal and Vertical Deviations

In this study, both student’s *t*-tests and multiple linear regression analyses of the horizontal and vertical deviations were negatively and significantly correlated with the improvement rates on the UPDRS-III sores. In addition, a lower stimulation voltage was used on our favorable group. The ear bar was inserted into both external auditory canals, which increased the symmetry of the head frame fixation. However, the ear bar insertion was stopped sometimes due to patient pain when the ear bar was adjusted to make the head frame more symmetrical. Therefore, manipulation of the ear bar resulted in a smaller difference in the vertical direction and a larger difference in the horizontal direction of the head frame fixation. The optimal diagnostic cut-off points for the horizontal and vertical deviations were 2 mm and 1 mm, respectively, which indicated that relatively favorable outcomes can be discriminated when the horizontal deviation was less than 2 mm or the vertical deviation was less than 1 mm.

### STN Length Indicated Favorable Outcome

Of the various pre- and intra-operative factors examined, the most important predictive factor for the clinical efficacy of STN stimulation was the length of the hyperactivity observed along the best track in the intraoperative multi-unit recordings (Lefaucheur et al., 2008). We also used intraoperative MERs to determine the length of the STN and optimal stimulation sites. Previous MER measures of the mean ± SEM length of the STN range from 4.9 ± 0.7 to 6.3 ± 0.5 mm (range, 2.5–6.9 mm) (McClelland et al., 2005; Kocabicak et al., 2013; Deffains et al., 2014). In our study, the mean ± SEM length of the STN according to the MERs was 5.37 ± 0.16 mm (range, 3.99–7.50), which was similar to previously reported values (McClelland et al., 2005). The spatial extent of the dorsolateral oscillatory region, which overlaps the motor territories of the STN, predicts the outcome of STN-DBS (Zaidel et al., 2010). Although the length of the STN is not proportional to the extent of the dorsolateral oscillatory region of the STN, the MERs showed that the length of the STN (within 4–6 mm) differed significantly (*p* < 0.001) between the favorable and unfavorable groups (Table 2). One possible explanation for this finding was that, when the frame was symmetrical, the trajectory of the recording lead was more accurate, which resulted in a favorable MER. Unfortunately, in addition to the symmetry of the frame, radio interference, anatomical variation of the DBS, and judgment differences of the operators could affect the MER measures of the length of the STN. In this study, we used the mean length of the right and left STNs in the analyses, and this might have decreased the differences in the statistical analysis. In future studies, the relationship between motor outcomes and the unilateral length of the STN should be analyzed according to the neural discharge rate in MERs, and these results may help to reconcile this problem.

### Postsurgical Peumocephalus-Associated Brain Shift

Several studies have reported that a few millimeters of brain shift at target regions can adversely affect the targeting accuracy during DBS surgery. This may occur as a result of cerebrospinal fluid loss, pneumocephalus, gravity-induced postural movements of intracranial structures, and/or brain deformation from the advancing electrode (Elias et al., 2007; Khan et al., 2008). The volume of postoperative pneumocephali, which are assumed to represent CSF loss, was significantly correlated with brain shift (Elias et al., 2007). However, several recent studies have revealed that brain shift has long been considered an issue in stereotactic targeting during DBS procedures and that subcortical brain shift is extremely limited and does not appear to adversely affect clinical outcome (Petersen et al., 2010; Slotty et al., 2012). Slotty et al. (2012) showed that intracranial air volumes up to 35 mL did not result in significant electrode displacement (Slotty et al., 2012). In our results, only one patient had a postoperative pneumocephalus over 35 mL (59 mL). Therefore, in our study, we used the postoperative pneumocephali volume to estimate the degree of brain shift. The rate of improvement in the UPDRS-III scores of the patients undergoing STN-DBS for PD did not differ significantly relative to the volume of postoperative pneumocephali.

### Study Limitations

This study is the first study to explore the impact of symmetric head frame on clinical outcomes following DBS surgery. The smaller case numbers from a single institution in this study might limit the generalizability of the findings. A multicenter study involving more cases should be performed in the future.

Another factor that should be taken into consideration is that the smallest quantified unit available within the frame system was 1 mm. Hence, the existence of an error of 1 mm in the three dimensions might have significantly impacted the prediction of STN location despite its relatively small size. To eliminate this error, the utilization of a more delicate frame system or robotic assistance may need to be explored. Severe skull deformities and/or variations in individual neuroanatomical structures may also impact the results. To achieve symmetric fixation, the depth and position of the head pin should be adjusted according to the shape of the skull. In some clinical situations, such as those involving hemimicrocephaly, the symmetrical fixation of head frames may not be possible.

In conclusion, this study showed that the indirect targeting of the STN in DBS with stereotactic CT guidance and MER was effective and safe for patients with advanced idiopathic PD. If these patients with PD have contraindications for MRI or cannot undergo MRI due to severe dystonia, obvious tremor, severely stooped posture, or claustrophobia, indirect targeting of the STN for DBS is a good choice. In addition, greater symmetry of head-frame fixations lead to better motor outcomes after subthalamic DBS in the patients with PD, especially when the horizontal deviation was 2 mm or less and the vertical deviation was 1 mm or less. In addition, the average length of the bilateral STN, which was between 4 and 6 mm according to the MERs, may be related to better motor outcomes.

## Author Contributions

P-HT, S-TL, and CL designed the experiment. P-HT and S-TL performed the surgeries. P-HT and Z-HL interpreted the data and wrote the manuscript. CC and WL performed the preoperative patient evaluations and intraoperative microelectrode recordings. CC and AB edited and proofread the manuscript. S-TL supervised the project and edited the manuscript.

## Conflict of Interest Statement

The authors declare that the research was conducted in the absence of any commercial or financial relationships that could be construed as a potential conflict of interest.
